# The effect of polyhexamethylene guanidine hydrochloride (PHMG) derivatives introduced into polylactide (PLA) on the activity of bacterial enzymes

**DOI:** 10.1007/s10295-014-1505-5

**Published:** 2014-09-05

**Authors:** Maciej Walczak, Agnieszka Richert, Aleksandra Burkowska-But

**Affiliations:** 1 Department of Environmental Microbiology and Biotechnology, Faculty of Biology and Environment Protection, Nicholaus Copernicus University, Lwowska 1, 87-100 Toruń, Poland; 2 Institute for Engineering of Polymer Materials and Dyes, Sklodowskiej-Curie 55, 87-100 Toruń, Poland

**Keywords:** Polylactide, Biocidal substances, PHMG derivatives, Activity of enzymes

## Abstract

The present study was aimed at investigating bactericidal properties of polylactide (PLA) films containing three different polyhexamethylene guanidine hydrochloride **(**PHMG) derivatives and effect of the derivatives on extracellular hydrolytic enzymes and intracellular dehydrogenases. All PHMG derivatives had a slightly stronger bactericidal effect on *Staphylococcus aureus* than on *E. coli* but only PHMG granular polyethylene wax (at the concentration of at least 0.6 %) has a bactericidal effect. PHMG derivatives introduced into PLA affected the activity of microbial hydrolases to a small extent. This means that the introduction of PHMG derivatives into PLA will not reduce its enzymatic biodegradation significantly. On the other hand, PHMG derivatives introduced into PLA strongly affected dehydrogenases activity in *S. aureus* than in *E. coli*.

## Introduction

In recent years, biodegradable polymer composites have become the subject of extensive scientific research [11]. This growing interest results from a wide range of their potential uses; medicine, biotechnology and packaging industry seem to be the most promising areas of their application [3]. The most popular of currently used polymers include polyhydroxybutyrate, chitosan, polycaprolactone, and polylactide [4]. Polylactide (PLA), already produced today on a large scale [10], is made by polymerization of lactic acid obtained through microbial synthesis from sugars found in plants such as corn and rice or through chemical synthesis from petroleum [11]. PLA properties have been thoroughly studied and described [12]. It is frequently accentuated that PLA is fully biodegradable, and the process, though slow, leaves no toxic products [19]. The biodegradation results from microbial colonization of PLA surface and the subsequent biofilm formation. This quality of polylactide, although favorable for environmental protection, is extremely disadvantageous for the food and medical industries. In food processing and medicine, materials whose surface can be easily colonized by microorganisms forming a biofilm are generally rejected. However, the introduction of biocides into the biodegradable polymer has proved to reduce microbial growth on its surface [6, 7, 20]. The term biocides (biocidal substances) denotes different microbiocides, sanitary, antiseptic or disinfecting agents employed to destroy harmful organisms. A range of substances, including natural and synthetic, organic and inorganic (sorbic acid, triclosan, grapefruit seed extract, chitinase, bacteriocin,) can be used for the production of materials with biocidal properties [8, 15, 27]. The great potential in the development of covalently bound permanent sterile surface possesses guanidine-based cationic polymers. PHMG and its derivatives exhibit extensive and excellent antimicrobial activity, also against antibiotics resistant bacteria [29].

Whereas, in general, the efficiency and operating range of biocides in their pure form have been sufficiently recognized, the efficiency of biocides introduced to polymers, mechanisms of their action and their impact on the natural environment still need more recognition [6]. It should be assumed that even highly biodegradable polymers will need more time to decompose after the addition of biocidal substances as no biofilm (which accelerates decomposition of the organic matter of polymers) will form on their surface [23].

Different biocidal substances have different modes of action, i.e., they affect different metabolic pathways and cell elements including hydrolytic enzymes secreted to hydrolyze the polymer. Impaired activity of these enzymes can lead to lower hydrolysis efficiency, which, in turn, impairs biodegradability of the composite. Another cell element, intracellular enzymes (dehydrogenases), involved in the process of cellular respiration, can also be negatively affected by a biocidal substance. A biocide inhibits respiration and impairs energy production in the cell.

The present study was aimed at investigating the effect of three PHMG derivatives on the activity of extracellular hydrolases and intracellular dehydrogenases.

## Materials and methods

### Materials

For the production of the investigated composite materials, we used biodegradable PLA polymer type 2002D (NatureWorks^®^, USA) with mass flow rate (MFR: 2.16 kg; 210 °C) of 3.4 g/10 min, and density (d) of 1.24 g/cm^3^ and the following bactericidal PHMG derivatives:

- PHMG granular polyethylene wax (W) (Institute for Engineering of Polymer Materials and Dyes, Poland).
- PHMG salt of sulfanilic acid (A) (Institute for Engineering of Polymer Materials and Dyes, Poland).
- PHMG stearate (S) (Institute for Engineering of Polymer Materials and Dyes, Poland).

Applied PHMG derivatives are compounds of a copolymer produced by PHMG synthesis of an organic carrier (polyethylene wax, sulfanilic acid, or stearate) according to Patent No: P.388062, 2009 [17]. The pelleted mixture of PLA and PHMG derivatives (PLA composites) was prepared with the use of the co-rotating twin-screw extruder type BTSK 20 (the diameter of the screws *L*′ 20 mm, *L*/*D* *L*′ 40) with a segmented plasticising system (Bühler, Germany).

The pelletising was performed in the form of a cool extrudate in the air with the temperature of 25 ± 3 °C. The extruded pellets were processed into sheets with the use of a single-screw extruder type PlastiCorder PLV 151 (Brabender, Germany) with a screw diameter of 19.5 mm and *L*/*D* = 25.

The samples had the following contents of PHMG derivatives: 0.0, 0.2, 0.6, and 1.0 % (wt). and were marked as follows (markings given in brackets): pure PLA (PLA), PLA with PHMG stearate (PLA-S), PLA with PHMG granular polyethylene wax (PLA-W) and PLA with PHMG salt of sulfanilic acid (PLA-A).

The mechanical and physical properties of the tested materials (tensile strength, water vapour permeability, mass flow rate) are described detail in an earlier publication [25].

#### Bacterial strains used in the research

The research was based on *Escherichia coli* ATCC8739 and *Staphylococcus aureus* ATCC 6538 strains.

### The assessment of bactericidal activity of PLA containing PHMG derivatives

Bactericidal properties of PLA containing PHMG derivatives were assessed according to standard ISO 22196 [14]. The analysis was performed in triplicate. Control (PLA) and test samples (PLA-S, PLA-W, PLA-A) covered with the suspensions of bacterial strains investigated in the research with a specified number of cells were left for a specified time (0 h—validation of recovery efficiency and 24 h). After this time, bacterial cells were recovered from the surface and suspended in a solution containing neutralizer. Subsequently, the number of cells capable of growth was determined by the inoculation on Plate Count Agar. The plates were incubated for 48 h at 37 °C.

Reduction of the number of living and viable cells of tested bacteria (*R*) was calculated using the equation:

$$R = (U_{t} {-}U_{o} ) - (W{-}U_{o} );$$

where *U*_*o*_ is the average of the common logarithm of the number of viable bacteria recovered from the control samples (PLA) immediately after inoculation (validation of recovery efficiency); the control samples (PLA) after 24 h (controls of survival in time, without PHMG derivatives);

*W* is the average of the common logarithm of the number of viable bacteria recovered from the test samples (PLA-S, PLA-W, PLA-A) after 24 h.

According to standard ISO 22196, the reduction of the number of cells capable of growth by two orders of magnitude (*R* ≥ 2) was interpreted as a bactericidal effect of the investigated composite.

### The assessment of microbial hydrolase activity

To separate the cells from the culture fluid, a 24-h broth culture of bacterial strains investigated in the research was centrifuged at 10,000 rev/min for 10 min at 4 °C. The obtained culture fluid containing hydrolases was used for further research. Samples of PLA films (5 cm× 5 cm) with 3 ml of the culture fluid were covered with smaller pieces of PLA films (4.5 cm × 4.5 cm) and placed in sterile Petri dishes. After pre-incubating the samples for 1 h at 4 °C, the culture fluid was collected into Eppendorf tubes. The general activity of hydrolytic enzymes after their contact with the PLA film was determined according to the procedure described by Adam and Duncan [1] using a non-specific substrate, fluorescein diacetate. The concentration of fluorescein released under the influence of hydrolases within 1 h at 30 °C was measured in triplicate using a spectrofluorimeter HITACHI F-2500, at an excitation wavelength of 480 nm and at an emission wavelength of 505 nm. The inhibition or stimulation (expressed as percent) was determined in relation to the control sample, i.e., PLA films without PHMG derivatives. The analysis was performed in triplicate.

### The assessment of dehydrogenases activity

The effect of PHGM derivatives on dehydrogenases activity was assessed using the TTC test, where triphenyltetrazolium chloride is reduced to triphenylformazan (TF). For this purpose, we prepared a bacterial suspension whose optical density OD = 2 according to McFarland corresponded to 6 × 10^8^ cells/ml. Then, fragments of PLA film (5 cm× 5 cm) with 3 ml of a reaction mixture contain the following elements: bacterial suspension (1 ml), Tris HCl buffer, pH 8.4 (1 ml), 2 % glucose solution (0.4 ml), 0.4 % TTC solution (0.4 ml), 0.36 % sodium sulfite (0.2 ml) were covered with smaller fragments of PLA films (4.5 cm × 4.5 cm), placed in sterile Petri dishes and pre-incubated at 4 °C for 2 h. The dishes were then transferred to 37 °C and incubated for 24 h. After the incubation, TF was extracted from the reaction mixture using *n*-butanol. TF concentration in relation to the standard curve was determined using a Hitachi U1900 spectrophotometer. The analysis was performed in triplicate.

### Statistical analysis

The results were statistically analyzed using two-way analysis of variance ANOVA, which allowed the comparison of two independent factors: the type of PHMG derivative and its concentration. The correlation coefficient between the concentration of PHMG derivative and its biocidal activity was also calculated.

## Results

### Assessing the bactericidal effect of PHMG derivatives introduced into PLA

**Fig. 1:**
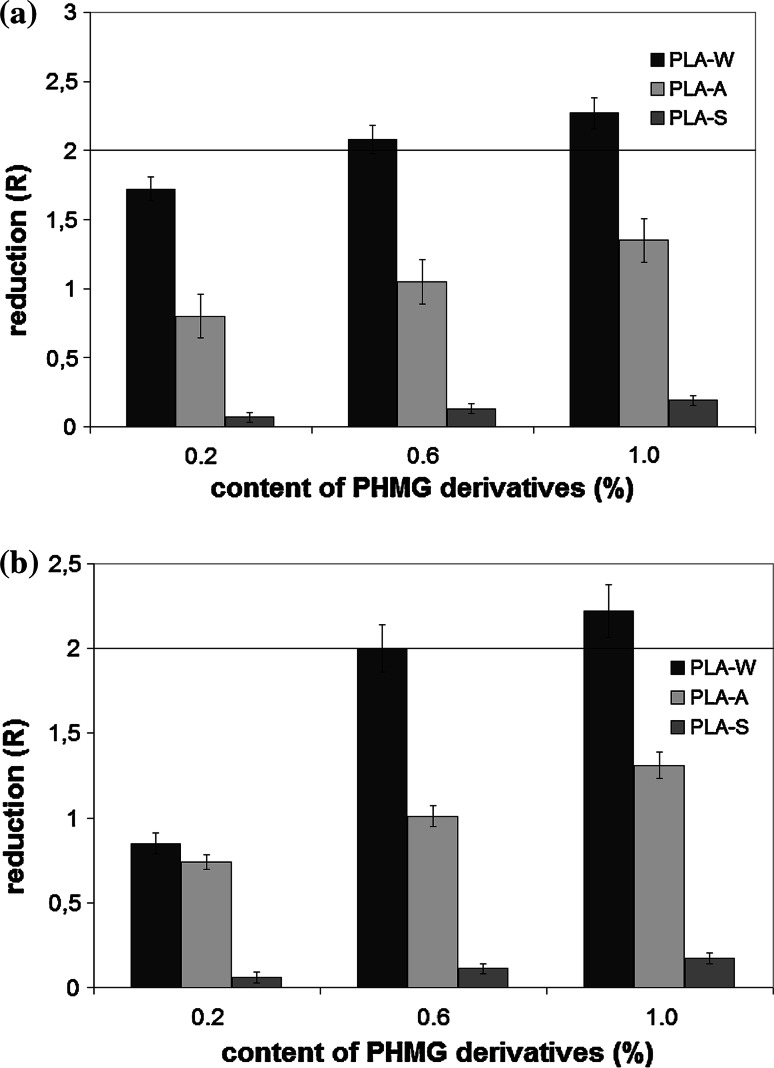
The bactericidal effect of PHMG derivatives introduced into PLA on tested bacterial strains (PLA-W granular polyethylene wax, PLA-A salt of sulfanilic acid, PLA-S stearate). According to standard ISO 22196, *R* ≥ 2 was interpreted as a bactericidal effect of the investigated composite. The data represented as ±SE from triplicate experiments. **a** *Staphylococcus aureus* **b** *Escherichia coli*

According to their bactericidal effect, PHMG derivatives used in the research can be put in the following order: PLA-W > PLA-A > PLA-S. The bactericidal activity of all PHMG derivatives was strongly positively correlated with their concentration (the correlation coefficient *r* ranging from 0.79 to 0.85). All PHMG derivatives had a slightly stronger bactericidal effect on *S. aureus* than on *E. coli*. According to ISO 22196, only PLA-W (at the concentration of at least 0.6 %) has a bactericidal effect. For both bacterial strains incubated on PLA films containing PLA-W at a concentration of 0.6 and 1 % for 24 h, there was a reduction of live and viable bacterial cells *R* ≥ 2 (Fig. 1).

### The effect of PHMG derivatives introduced into PLA on the activity of microbial hydrolases

**Fig. 2:**
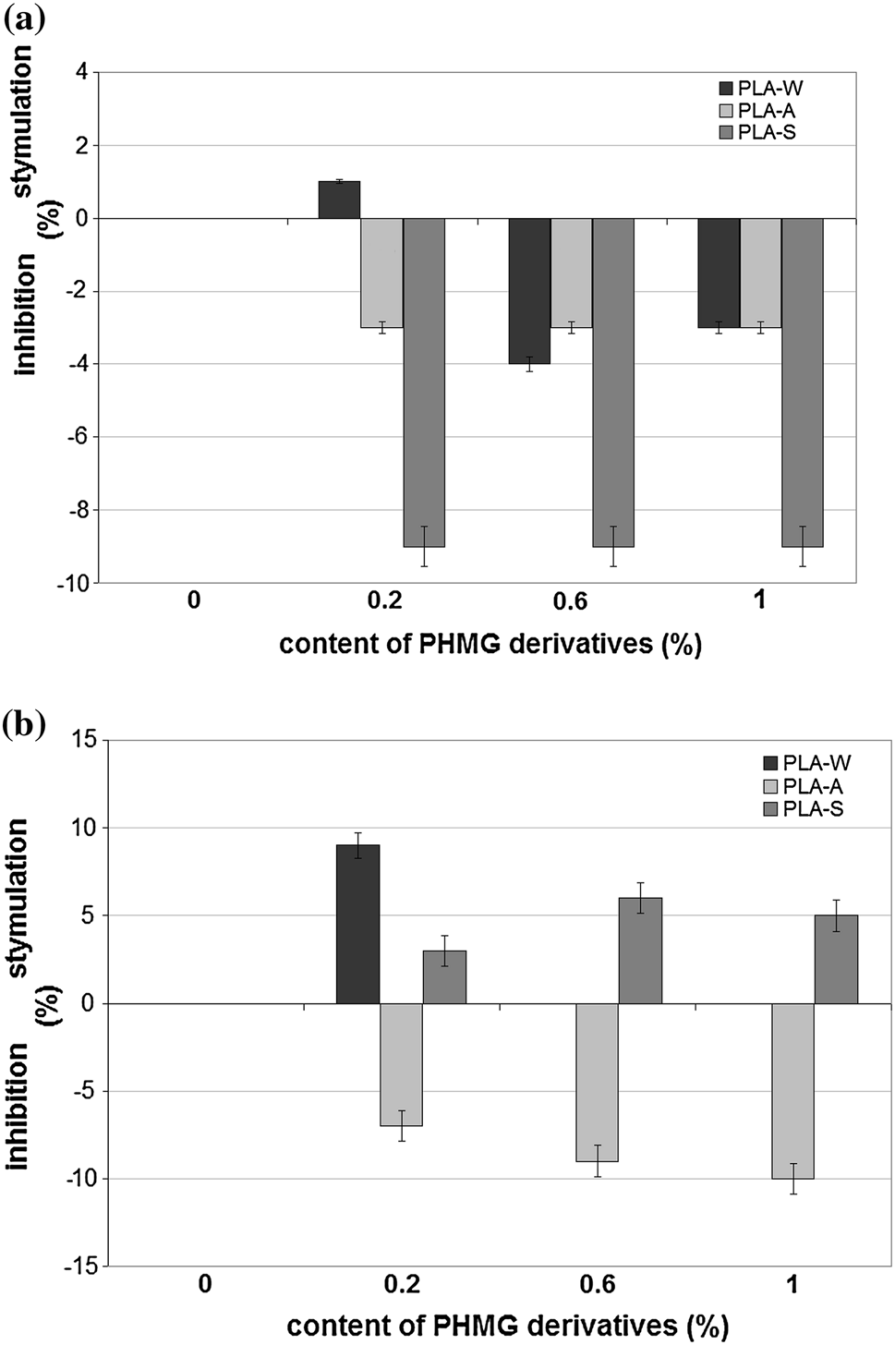
Effect of PHMG introduced into PLA on hydrolase activity (PLA-W granular polyethylene wax, PLA-A salt of sulfanilic acid, PLA-S stearate). The data represented as ±SE from triplicate experiments. **a** *Staphylococcus aureus* **b** *Escherichia coli*

PHMG derivatives introduced into PLA affected the activity of microbial hydrolases to a small extent. In *S. aureus*, they inhibited hydrolase activity by approximately 9 %. However, statistical analysis revealed no significant differences in hydrolase activity related to the concentration and type of PHMG derivatives (Fig. 2a). PLA-S composite with PHMG stimulated hydrolase activity in *E. coli*, but the differences were not statistically significant (*p* < 0.1). PLA-A composite slightly inhibited hydrolase activity in *E. coli*, proportionally to the concentration of PHMG, but the differences were not statistically significant (*p* < 0.06). PLA-W composite with PHMG did not affect hydrolase activity in *E. coli* (Fig. 2b).

### The effect of PHMG derivatives introduced into PLA on dehydrogenases activity

**Fig. 3:**
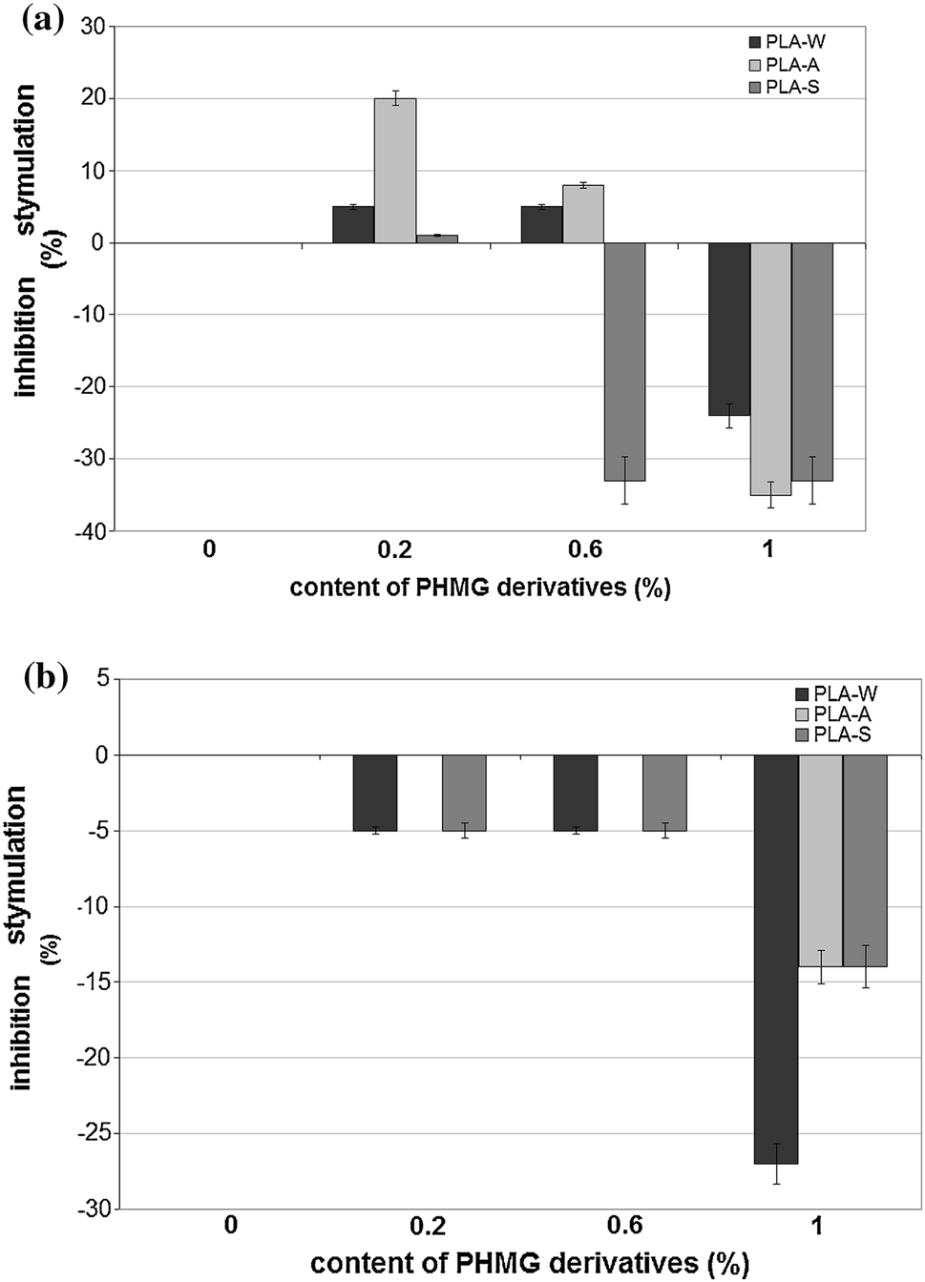
Effect of PHMG introduced into PLA on dehydrogenase activity (PLA-W granular polyethylene wax, PLA-A salt of sulfanilic acid, PLA-S stearate). The data represented as ±SE from triplicate experiments. **a** *Staphylococcus aureus* **b** *Escherichia coli*

PHMG derivatives introduced into PLA strongly affected dehydrogenases activity in *S. aureus*. All PHMG derivatives at highest concentration (1 %) significantly inhibited dehydrogenases activity in this bacterial strain (*p* < 0.001) (Fig. 3a). In contrast, PHMG derivatives had significantly smaller effect on dehydrogenases activity in *E. coli* (Fig. 3b). Only PLA-W significantly inhibited dehydrogenases activity in this bacterial strain (*p* < 0.022). Other PHMG derivatives inhibited dehydrogenases activity only to a small extent or failed to affect dehydrogenases activity.

## Discussion

Guanidine derivatives have been used as bactericides and fungicides for many years. Owing to its broad spectrum, bactericidal and fungicidal activity, polyhexamethylene biguanide (PHMB) has been one of the most popular [22]. Unlike PHMB, polyhexamethylene guanidine (PHMG) is a relatively new compound whose properties, potency, and effects are not yet fully recognized. Hitherto studies have shown that PHMG in solutions has fungicidal activity [9, 24] as well as bactericidal activity against Gram-positive and Gram-negative bacteria [16, 28, 29].

The results of the present study indicate that PHMG derivatives introduced into a biodegradable PLA polymer inhibit microbial growth on its surface. The strongest bactericidal effect was observed for PLA-W, which at concentrations of 0.6 and 1.0 % proved bactericidal according to ISO 22196 [14]. Other PHGM derivatives reduced the bacterial survival rate, but their effects were too weak to be considered bactericidal according to ISO 22196 [14]. At the same time, other researchers investigated bactericidal activity of PHMG introduced into polyvinyl alcohol (a synthetic polymer) at concentrations of 1 and 5 % [21]. The results indicated inhibited growth of *E. coli* but no bactericidal effect was observed according to ISO 22196 [21]. The authors accentuated the reduced adhesion of *Pseudomonas aeruginosa* to the surface of the investigated composite, which indicates inhibited biofilm formation caused by the addition of PHMG [21]. Similar results were obtained by Richert et al. [25].

Although biocidal activity of PHMG inhibiting biofilm formation on polymers such as PLA is extremely valuable for the packaging industry and medicine, it can significantly reduce the biodegradability of polymers generally considered environmentally friendly [5, 23]. Hence, the present research focused primarily on the effect of PHMG derivatives introduced into PLA on the activity of bacterial hydrolytic enzymes and cellular dehydrogenases.

The results indicate that PHMG derivatives affected hydrolase activity to a small extent and the changes were not statistically significant. This finding is of extreme importance because, as has been already demonstrated, PLA biodegradation results primarily from the activity of extracellular enzymes, mainly serine proteases belonging to esterases [18] and lipases [2, 13]. The inhibition of enzymes classified as so-called PLAases, participating in PLA biodegradation, significantly reduces the biodegradability of this biopolymer [18]. PLAases inhibition would mean that PLA with PHMG derivatives would not be considered a ‘green polymer’.

The results also indicate that PHMG derivatives introduced into PLA inhibit dehydrogenases activity, particularly in *S. aureus*. However, only PLA-W had the inhibitory effect on *E. Coli*. Dehydrogenases, belonging to the group of Oxidoreductases, are found as a complex at the plasma membrane [26]. A number of studies indicate that bactericidal activity of PHMG involves damaging the cell membrane [9, 28]. The results of the research by Feng et al. [9] and Zhou et al. [28] demonstrate that PHMG not only affects permeability of the cell membrane, but also, and most importantly, damages the cell membrane mechanically, causing the leakage of the cell contents to the external environment, and the subsequent cell death [28]. In addition, Zhou et al. [28] indicate that the potency of the PHMG depends on its concentration, exposure time and the size of the bacterial inoculum. The authors [28] also show that PHMG damages the outer membrane first (if a bacterium has one), and only at higher concentrations damages the cell wall as well. This observation suggests that the presence of the outer membrane increases the resistance of some bacterial cells to PHMG. These results are consistent with our observations indicating a strong correlation between the concentration of PHMG derivatives and their bactericidal activity and explain the stronger bactericidal effect on *S. aureus* (which lacks an outer membrane) than on *E. coli* (which have an outer membrane).

Inhibiting the activity of cellular dehydrogenases by PHMG may significantly inhibit microbial biofilm formation on the surface of the composite. Although considered advantageous for the application in industry or medicine, this quality can negatively affect the biodegradation of biopolymers containing PHMG derivatives. Enzymes secreted by microorganisms and microbial biofilm formation are responsible for PLA biodegradation [23].

## Conclusion

PHMG derivatives introduced into PLA reduced viability of bacteria *E. coli* and *S. aureus*. However, only PLA-W (PLA with PHMG granular polyethylene wax at concentrations of 0.6 and 1.0 %) had bactericidal activity according to standard ISO 22196, which means that the composites produced from PLA and this PHMG derivative can be used in many areas (including the packaging industry) to reduce the growth of microorganisms.

At the same time, the investigated PLA composites did not inhibit the activity of extracellular hydrolytic enzymes, which means that the introduction of PHMG derivatives into PLA will not reduce its enzymatic biodegradation significantly. The results indicate that PHMG derivatives inhibited the activity of cellular dehydrogenases, the fact which may be related to PHMG mode of action, relying primarily on damaging the cell membrane.
